# Religion, Culture and Meaning-Making Coping: A Study Among Cancer Patients in Malaysia

**DOI:** 10.1007/s10943-018-0636-9

**Published:** 2018-06-13

**Authors:** Fereshteh Ahmadi, Nur Atikah Mohamed Hussin, Mohd Taufik Mohammad

**Affiliations:** 1 Department of Social Work and Psychology, Faculty of Health and Occupational Studies, University of Gävle, 801 76 Gävle, Sweden; 2 Social Work Section, School of Social Sciences, Universiti Sains Malaysia, 11800 Pulau Pinang, Malaysia

**Keywords:** Meaning-making coping, Malay cancer patients, RCOPE (religious coping methods), Spiritual-oriented coping, Existential coping methods, Culture and coping

## Abstract

The present study aimed to explore the use of meaning-making coping mechanisms (existential, spiritual and religious coping) among ethnic Malay cancer patients in Malaysia and to investigate the impact of culture on their choice of coping methods. Twenty-nine participants with various kinds of cancer were interviewed. Four kinds of coping resources emerged from analyses of the interview transcripts: (1) relying on transcendent power, (2) supernatural or mystical beliefs, (3) finding oneself in relationships with others and (4) nature. In this article, the two first resources are in focus. The present findings suggest that Malay culture, which is imbued with Islamic belief, strongly influences cancer patients’ coping methods and ways of looking at their experience of being cancer patients.

## Introduction

Cancer remains a threat among the Malaysian society nowadays. According to the Malaysian National Cancer Registry Report (MNCR) (2007–2011), there were 103,507 new cancer cases reported from 2007 to 2011. Females made up 46,794 cases (54.8%) of these reported cases, while males 56,713 cases (45.2%).

It has been reported that, in Malaysia, cancer patients find it difficult to cope with their stress after the diagnosis and choose to commit suicide at the hospitals where they reside (Satibi 2015). Satibi also reported that, from 2008 to 2013, there were 50 cases of patients who jumped from high floors in public hospitals in Malaysia and that 31 cases resulted in death. The National Suicide Registry of Malaysia (2009) reported that 11.4% of the total suicide cases in 2009 were victims with a history of cancer diagnosis.

A few studies have reported on the relationship between level of religiosity and its effect on suicide (e.g., Dervic et al. 2004; Wu et al. 2015). Given that Malaysia is a highly religious society (Gallup International 2012), it might be supposed that religion plays an important role in alleviating the experience of being a cancer patient, but the statistics compiled above raise the question of why these suicidal cases still occur (Inglehart et al. 2010).

Confronting a life-threatening disease such as cancer can be very stressful. In order to cope with the experience, cancer patients may search for meaning in relation to why they got cancer. Assigning meaning in life is vital, as it seems to impact psychological well-being and may help to moderate negative feelings, especially after being diagnosed with cancer (Tomich and Helgeson 2002). Some researchers have maintained that religiosity and spirituality are the main factors facilitating meaning-making coping. However, more recent studies by Ahmadi (2006, 2015) have shown that, apart from religiosity and spirituality, cancer patients were able to use other meaning-making resources, such as nature, to help them to find some meaning in their illnesses.

Searching for coping methods to find meaning has been described as having the potential to bring positivity into individuals’ lives, especially after a cancer diagnosis (Schroevers et al. 2011). In addition, the ability to find meaning after the diagnosis helps cancer patients create new goals in life. In this way, cancer patients are able to make the necessary adaptation to their illnesses, while still preserving hope about the future.

However, other studies have associated the search for meaning after the diagnosis with poorer adjustment (Roberts et al.’s 2005). This study revealed that the process of searching for meaning could be also associated with intrusive thoughts that make it difficult to adjust to the situation cancer brings about. In addition, cancer survivors or patients who are unable to successfully integrate their beliefs into the process of searching for meaning may also reflect on their cancer experience without achieving any resolution (Holland and Reznik 2005).

## Theoretical Background

Being diagnosed with cancer may affect various domains in the patient’s life: physical, psychological, interpersonal, vocational and spiritual (Aziz 2007; Hewitt et al. 2003). A cancer diagnosis may also affect the patient’s daily functioning, for example, by limiting her/his ability to work. Young cancer patients seem to be at a higher risk of experiencing psychological problems, which may extend over time (Arndt et al. 2004; Costanzo et al. 2009; Hewitt et al. 2003). These psychological problems may include young cancer patients being unable to lead healthy lifestyles, which may prevent them from achieving positive quality of life in the long term.

After diagnosis, patients may employ various methods to cope with their experience of being cancer patients and to maintain positivity. One of the coping styles that have received a great deal of interest among researchers involves searching for meaning (e.g., Lee et al. 2004; Martino and Freda 2016; Tomich and Helgeson 2002). A searching for meaning coping style is referred to as accommodative coping, which involves cognitive restructuring. According to Folkman and Moskowitz (2000), there are two forms of meaning making: situational and global. Situational meaning refers to appraisals of stress that help determine the degree of personal significance of the tragedy in relation to a person’s beliefs, goals or values. Global meaning, on the other hand, is more concerned with the abstract, generalized meaning that is related to people’s existential assumptions or “assumptive worlds” (Janoff-Bulman, 1992).

Folkman and Moskowitz (2000) found positive effects of searching for meaning among people who suffer from chronic diseases. Searching for meaning cultivates positive emotions and improves well-being. Positive emotions gained from the process of making meaning help broaden an individual’s thought and action, thereby building his/her enduring personal resources and counteracting negative emotions (Fredrickson et al. 2000). Studies found that religious belief and spirituality may also facilitate the process of searching for meaning among cancer patients, helping them make sense of their illnesses (e.g., Jenkins and Pargament 1995; Johnson and Spilka 1991). Having a high level of religiosity and spirituality causes cancer patients to believe in a higher power and to see the situation as being “part of a bigger plan” or as a sign of God’s love. However, Ahmadi (2006) revealed that there are also other resources for meaning making that help cancer patients who do not believe in God make sense of their situation (Ahmadi 2006). Ahmadi’s study of 2355 Swedish cancer patients showed that nature provided strength and tranquility for them to deal with their illnesses. This finding suggested a new method of coping, which was defined as “new spiritually oriented coping methods dissimilar to religious coping.” In this article, we focus, however, only on religious coping methods, not these new ones.

## Meaning-Making Coping

Coping is often considered a multilayered contextual phenomenon (Lazarus and Folkman 1984: 148; Pargament 1997: 89)—a process individuals undertake to try to understand and manage important demands in their lives (Ganzevoort 1998: 260), or a quest to find or create meaning in life during periods of stress (Pargament 1997: 90). Furthermore, coping has been suggested to be a process that develops and changes over time (Pargament 1997: 89).

Earlier studies have used the terms “religious” and “spiritual” to refer to coping methods that are principally based on existential issues (Ahmadi 2006, 2015). Findings from previous studies (Ahmadi 2006, 2015; Ahmadi and Ahmadi 2015), carried out among people who lived in predominantly non-religious contexts and had been struck by cancer, indicate that other coping strategies are frequently employed in these contexts—strategies that can hardly be considered religious or spiritual. Among these strategies are Spiritual Connection with Oneself, Spiritual Sanctification of Nature, Positive Solitude, Empathy/Altruism, Search for Meaning, Visualization, Healing Therapy, Spiritual Music and Meditation. An analysis of these methods clearly showed that they had much more to do with *connectedness to nature, to self and to others than to something transcendent (God or any spiritual power) (*Ahmadi 2006).

Although the term “existential coping methods” may still be used, given that these methods concern people’s efforts to find a source—in nature, in themselves or in others—that could help them cope with their problems, we wish to avoid restricting ourselves to either the religious or secular nature of these methods and will hereafter use the term “meaning-making coping.” Meaning-making coping is thus used in the present study to address the whole spectrum of religious, spiritual and existential coping methods.

### Background of the Ethnic Malay Community

Malaysia is a multiracial and multi-religious country in South-East Asia. The Malaysian citizens comprise the Malay (68.6%), Chinese (23.4%), Indians (7%) and other (1%) ethnic groups (Department of Statistics Malaysia 2016). In terms of religions, Islam, the official religion of the country, is the most widely professed religion in Malaysia, followed by Buddhism (19.8%) and Hinduism (6.3%). However, all of the religions are free to be practiced in peace and harmony, as stated in the Federal Constitution of Malaysia (Mohd Sani & Abdul Hamed Shah 2011). However, Malaysia does not tolerate any denominations of Islam other than Sunni Islam. Any teaching that deviates from the official Sunni code is illegal, and no other forms of Islam are allowed (International Religious Freedom Report 2016).

Islam has a great influence on various aspects of the lives of the Malay people (Oka et al. 2017). Compared to other Muslim populations in the world, the Malay Muslims have “unique” characteristics. In understanding an illness medically, besides the scientific explanation, Malay people also believe in supernatural and mystical aspects of illnesses. This is due to the influence of animistic belief, which preceded Islam (Haque, 2005). Some Malay people believe that any illness they have is caused by unexpressed *angin* (wind) that gets stuck in the body and induce pathology (Haque 2005; Ng et al. 2003). *Angin* is believed to be a whirl of wind that gets stuck in some parts of the body and that causes discomfort. Some also believe in supernatural causes, such as the possession of *Jinn* (Genie) or the result of *Santau* (black magic), typically sent by enemies (Haque 2005).

Such belief opens up another possibility for Malay people to get treatment. They may choose either a conventional medical doctor or a traditional healer, called a *pawang* or *bomoh,* to get rid of any spiritual possession that may cause their physical and psychological illnesses.

Among the three major races in Malaysia, namely the Malay, Chinese and Indian, evidences found that Malay cancer patients were prominent to be diagnosed at a later stage and to have poorest prognosis (Taib et al. 2008; Xin et al. 2017). A qualitative study on female Malay with advanced breast cancer reported that the patients were most likely to discontinue their treatment and cope with their illnesses by turning to spiritual belief to fight with their cancer (Ahmad et al. 2011). Nevertheless, our knowledge of how Malay people cope with their illnesses is still limited. Previous researchers in other countries had discovered new coping mechanisms—such as Sanctification of Nature, Spiritual Connection with Oneself, Positive Solitude, Altruism, Spiritual Music (Ahmadi 2006, 2015)—which may or may not be prevalent among Malay cancer patients. According to Ahmadi (2015), these coping mechanisms are described as a part of meaning-making coping which may not related to religion only but also influenced by everyone’s cultural belief. A study among Korean cancer patients in Korea, for example, reported that their belief on nature as a healer has helped the Korean cancer patients to cope with their cancer better (Ahmadi et al. 2016). Therefore, the present study aims at deepening our understanding of how Malay cancer patients use the elements of religiosity and spirituality to cope with their illness. The study also aims to integrating the role of culture in choosing different meaning-making coping methods.

### Methodology

Approximately 35% of the Malaysian population (37.1 million) was reported to use Facebook in 2013 (Mahadi 2013). Based on this, and to further enhance the voluntary nature of participation, the researchers used Facebook as a medium to recruit respondents from various locations and age groups in Malaysia. Twenty-nine participants were recruited to the present study by posting a participant recruitment advertisement in a closed Facebook group where cancer patients/survivors could post a comment indicating their intention to voluntarily participate in the study. In the first steps, only Muslim informants were chosen.

The participants were contacted using the contact information left on the Facebook advertisement. Although Facebook users in this closed group have been monitored by several administrators, the researchers also applied a strict verification process before the interview, where potential participants were asked to verify their status by sharing documents related to their medical and treatment histories. The documents were then verified by an appointed oncologist. After verification, the researchers contacted the potential participants by phone to make an appointment for the interview.

Interview questions for the study were primarily based on results from the Swedish study (Ahmadi 2015). The original interview guide was translated from English to the Malay language by two Malaya researchers involved in the project. The questions were modified to better suit the Malay culture.

## Procedure

Prior to the interview, the researcher obtained the participant’s informed consent. In the present study, all respondents’ names were kept anonymous. The location and time of the interviews were chosen according to the participants’ preference. Each interview lasted from around 45 min to one and half hours. Bahasa Malaysia was spoken during the interviews.

The data were obtained from 29 Malay cancer patients between 29 and 60 years of age. Their respective cancer survival longevity ranged from one to 25 years. Their level of education varied from high school to Doctor of Philosophy (PhD). Participants’ stage of cancer varied from earliest, palliative care and survivors. The participants’ backgrounds varied from housewife, retirees, businessman, working in private or government sectors and educators. All of the participants are Sunni Muslims. The socio-demographic background of the respondents is summarized in Table 1.

**Table 1:** List of demographic characteristics of the participants

Themes	Categories	*N*
Gender	Female	21
	Male	8
Age	25–30	2
	31–40	15
	41–50	9
	51–60	3
Education	High school	7
	Undergraduates	17
	Postgraduates	5
Employment status	Housewife	6
	Businessman	3
	Private sector	5
	Government sector	5
	Retirees	2
	Educators	8
Survival longevity	0–5 years	23
	6–10 years	2
	11–20 years	3
	21–25 years	1

The interviews were fully transcribed and translated into English. A professional translator was used to ensure the accuracy of the translation. Coding, identification of the themes that emerged and identification of the relationship for each theme were done during the analysis process. Codes were grouped into categories. The research team identified, discussed and finalized the categories and themes that emerged from the interviews. An expert on cultural backgrounds has also been consulted to ensure the validity of the analysis.

Achieving an acceptable degree of the trustworthiness, which guaranties the credibility of qualitative research, is an important issue in qualitative research (Morrow 2005). There are four general types of trustworthiness in qualitative research: credibility, transferability, dependability and confirmability (Lincoln and Guba 1985). To achieve credibility, the researcher developed a rapport with the participants. They were also told to answer the questions honestly without thinking about searching for the “correct” answers. To achieve credibility, the researcher explained the rationale of the study and shared stories about self-experience to promote a comfortable and trustful relationship between the participants and the researcher. To ensure validity, each answer given by the participants was paraphrased by the researcher, and the participants were asked to listen and validate the statement. To establish transferability, the interviews were conducted with care to ensure that every item in the interview guide was covered. To establish dependability, the researcher carefully discussed the procedure, process and analysis to establish inter-subjectivity. Finally, to establish confirmability, the researcher applied bracketing, which entails excluding one’s own perceptions and experience throughout the research activity.

## Results

The 29 respondents interviewed each told their own story. In the present article, we only touch on the first two themes, which mainly concern the religious coping methods. In presenting the results, we proceed from the RCOPE categories and themes.

### Theme 1: Religious Coping Methods

In order to categorize these methods, we used the five key religious functions that constitute the basis of RCOPE.

#### Category 1: Find Meaning

Sub-categories

##### *Benevolent Religious Reappraisal*

using religion to redefine the stressor as benevolent and potentially beneficial.

We observed a coping method, used by some informants, that can be categorized as Benevolent Religious Reappraisal. We found two patterns. The first pattern refers to cancer as a lesson from God. In the other pattern, like in the story of Job, suffering is seen as educational theodicy (Dein 1997). Concerning the first pattern, one interviewee—a 41-year-old man, who admitted that before being struck by cancer, he felt neither close to God nor close to his children—redefined the situation as follows:

I believe that among the reasons why God gave me this illness is that He wants me to be closer to Him…… I become stronger and open to accept everything… I now spend more time with my kids. I always bring them to nature and ask them to think: “look at the nature. This is all God` s power. You must be thankful; God gave me more time to spend with you. If I die, you have no one. So be thankful”.

A 37-year-old man, who had used the same method, told us that:

Honestly, I was not a good person before. I did not pray, I did not go to the mosque, I was not even close to my family. My life was not blessed [by God]. No matter how rich I was, I still felt like it wasn’t enough. I had already undergone the dark time when I was sick. The moment I was sick, it became my turning point. From the dark time until now, thank God, there is a bit of light. … Maybe other methods would not be enough to make me aware, but when God gave me this sickness, it reminded me of death. I considered it as a time for me to give back to others….Cancer is my turning point, I guess….Now I spend more time with my family. We go for vacation and I am thankful to God. Some people they do not know that they are doing the wrong thing until they pass away, but I got a chance to change for the better.

Using the coping method Benevolent Religious Reappraisal, the individual tries to find a lesson from God in the event or to see how the situation could be spiritually beneficial. As the above examples show, for some interviewees the illness is regarded as a turning point. They regard cancer as a gift from God—a gift that helps them see their life in a new light, which leads them to God and their family; in doing this, they can better deal with the stressor and feel relaxed. In other words, being struck by cancer caused them to reexamine their past life and find a lesson there.

The second pattern is “educational theodicy.” Regarding illness as a test that God imposes on us is an old idea. Illness may be seen as the result of God’s will and therefore accepted or, as in the story of Job, seen as educational theodicy (Dein 1997). In this regard, a 65-year-old woman told us:

Why does God give this to me and not to other people? So God wants to elevate our status, then the miracle that Allah wants to give.

Another interviewee, a 39-year-old man, emphasized:

This is a test for me. I just surrender myself to God and I believe in *Qadar* [destiny]. I always think positively, every human will die. Are we ready? God gives us warning in different ways.

A 41-year-old woman explained:

I just think about one thing; this illness is a gift from God so that I remember Him more. Maybe I always forgot Him before this.

Another interviewee, a 33-year-old woman, clarified:

I consider this illness a test from God to test my faith. At the same time, my sins can be eradicated; those which I have forgotten or have not been aware of. The wisdom is that God still loves me by giving me a chance to be closer to Him.

As these citations show, the interviewees regard their illness as what is called “educational theodicy” (Dein 1997), which means that God allows suffering to test the person and then promote her/him a better life, as in the story of Job. Suffering helps the person appreciate the good things in life, and therefore to grow.

##### *Punishing God Reappraisal*

redefining the stressor as a punishment from God (sins I have committed in my life):

A 40-year-old woman emphasized: “I always think that this illness is because of my sin, but I need to be strong to see my kids grow up.” Another interviewee, a 41-year-old man, explained: “I believe that I got cancer because of my sin and my habit.”

Ahmadi (2006:106) suggested that applying Punishing God Reappraisal as a coping method “presumably requires a belief in a God who can determine the course of individuals’ lives: a God who not only created man, but also continually controls man`s deeds and his destiny.” Among many Muslims, the prevalent notion of God tends toward this view. In this connection, Aflakseir and Coleman (2011:46) emphasized “Islamic teachings encourage people to be patient, to perform prayer, and to trust and to turn to God in times of need and for guidance.” They also explained that, according to the Qur’an and Islamic traditions, one way of reaching a state of improved well-being and being able to cope with difficulties is to “remember the Name of your Lord and devote yourself with a complete devotion” (Qur’an verses 73:8).

##### *Demonic Reappraisal*

which refers to redefining the stressor as an act of the “Devil”/an evil power—is another coping method used in this group.

We found several informants who believed in *Black magic* and therefore used the coping method of Demonic reappraisal.

A 33-year-old woman answered the question concerning the demonic reappraisal coping method as follows:

Yes. I went to seek spiritual treatment, I saw a religious person …. There was someone who sent me the negative thing, and it was shown to me. What the motive is I don’t know.

Another interviewee, a 35-year-old woman, explained:

Black magic; Yes, I was told that this is sent by people. He cursed me and I got cancer. I went everywhere to get help. Started with the fact, added forgetfulness, taking things for granted and at last, fear and trauma became my own flesh.

Black magic or dark magic has traditionally referred to the use of supernatural powers or magic for evil and selfish purposes. The idea of black magic and Shamanism in Southeast Asia can be traced to the region’s prehistoric tribal people. However, Muslim scholars regard the practice of shamanism as *shirk* (idolatry, deification of figures other than Allah). What we see here is the impact of the culture on health beliefs, which are stronger than fundamental religious axioms. We will return to this point in the discussion section.

#### Category 2: Gain Control

Sub-categories

##### *Passive Religious Deferral*

Passively waiting for God to control the situation.

Some interviewees seek comfort and cope with the stressor through *Passive Religious Deferral*.

A passive approach to facing one’s illness and totally relying on God was found.

One 40-year-old woman explained this as follows:

Nature is not helping me to gain calmness but it makes me think that everything that happened is a plan by God.

Another interviewee, a 60-year-old woman, told us:

When I got cancer, I just thought that it was Allah who gave it to me. I just prayed to Allah….. I just surrender. … So now I just focus on praying.

A 35-year-old woman emphasized:

Before we were born, we already had an agreement with God. God told you who you would be married to, anything. Do you agree? If you agree, your soul is breathed into the fetus. My life is only borrowed.

One 32-year-old man also believed in the agreement idea. He believed the following:

Actually, before we were born, we made an agreement with God. We agreed to be born like this. Therefore we have to accept everything that happened to us.

Here we are dealing with the idea of *Qadr (Kader),* which means “fate” or “predestination.” In Islamic thought, *Qadr (Kader)* refers to divine destiny. Some Muslims believe that God wrote down in the *Preserved Table* (“al-Lauḥ al-Maḥfūẓ”) everything that has happened and that will happen. This means that everything that occurs in the world (cosmos) has been dictated by God and based on his prior knowledge and states of wisdom.

The idea of testing (*Ekhtebar*) and being patient (*Sabr*) is quite strong among Muslims. According to Aflakseir and Coleman (2011:46):

The Qur’an emphasizes clearly that the difficulties in this world are to test the believer and also asking people to have patience in facing their problems. For example, the Qur’an says: ‘We try you by means of danger, and hunger, and loss of worldly goods, of lives and of [labor’s] fruits, But give glad tidings unto those who are patient in adversity’ (2:155). Therefore, according to the religious teachings, the negative events have a purpose and people are required to be patient to achieve spiritual growth.

The idea that the problems of this world are put there to test people and encourage them to have patience when facing problems is, as it seems, strong among people in Muslim countries, including Malaysia.

##### *Active Religious Surrounding*

actively giving up control to God (I did my best and now I am relying totally on God).

In this connection, a 65-year-old woman explained:

I tried all of the treatments but if God says that she will die, treatment is an effort, if God permits us to be healthy, we will be healthy. If not, our age stops here.

This interviewee explained to us that she has handed over control to God after having done her best and having undergone various treatments. Here, although we see an Active Religious Surrounding method, there is also a trace of belief in fate, or *Kader* in Islam.

##### *Pleading for Direct Intercession*

seeking control indirectly by pleading to God (praying)

A 36-year-old woman describes using this method as follows:

I try to find the tranquility by being closer to Allah, performing prayers, doing remembrance, reading the translation of Qur’an and seeking for religious knowledge.

Here, the informants tried to gain comfort by pleading to God to make things turn out positively.

##### *Collaborative Religious Coping*

seeking control through a partnership with God

Regarding this coping method, one interviewee, a 59-year-old man, explained:

A Wednesday morning, when it was time for my operation, I woke up to pray, then I communicated with myself. ‘Karim, what happened to you? You are sick.. This operation is a an attempt to cure me’…..After the operation, I relied mainly on myself…. …So my problems turned into challenges….. I became stronger. I looked at what I underwent as a challenge, not an obstacle. I am also closer to God.

When they use this method, the informants are not being passive and relying on God to help them, but they are also relying on their own power. The interviewee quoted above did not only plead for miracles; he also relied on himself and physicians. What we see here is a collaboration between the patient, physicians and God. God is given the role of a partner. God lends his hand not to pull the patient out of a difficult situation, but offers a hand the patient can hold to get through the situation.

In general, with regard to the RCOPE category Gain Control, we can maintain that the ideas of *Qadar (*Kader) or faith and *Sabr* (patient) were predominant among the interviewees. The interviewees have shown a tendency toward relying mainly on God; regarding their illness as fate caused them to realize that they should have patience and that patience would have certain benefits.

#### Category 3: Gain Comfort and Closeness to God

Sub-categories

##### *Religious Purification*

searching for spiritual cleansing through religious actions (visiting the Mosque, reading religious texts)

One 36-year-old woman described using this coping method as follows:

I also found Qur’anic verses and remembrances that can be practiced; they calmed down my emotions and helped to heal this illness.”

Another interviewee, a 35-year-old woman also sees reading Qur’an as a way to cope with her illness:

I allowed myself to cry and grieve for two weeks. Then I started to build up my spirit. I do it one step at a time. I did the closed-door policy. So that time no one was allowed to tell a story about which people die from cancer. And when I felt hopeless, I read Qur’anic verses, especially, sura Al-Inshirah.

The Al-Inshirah is a famous *sura* (passage) in the Qur’an. It informs Mohammad (the prophet) that his burden and difficulties, which he endures owing to his prophetic mission, will soon be removed. In fact, the massage says that with each difficulty there is ease. This passage has attracted the interviewee quoted above, and probably many other Muslims facing serious problems. People read it and get comfort by believing that God will lift their burden, as he once promised he would do for Mohammad.

Muslims consider the Qur’an to be Islam’s miracle and the direct words of God; for some Muslims, reading it is an integrated part of everyday life. Thus, it is quite understandable that these interviewees use *Religious Purification* as a coping method and that they try to cope with their illness by searching for spiritual cleansing through reading Qur’an.

Reading religious texts is not a passive approach to facing a crisis. On the contrary, it is an active attempt to understand what one is going through and to place one’s experiences in a comprehensive context. In cultural settings where prayer has been an integrated part of everyday life, people who are facing a crisis readily turn to reading religious texts. Here, reading is a way to control emotions and overcome fear and anxiety.

#### Category 4: Gain Intimacy with Others

Sub-category

##### *Seeking Support from Clergy or Congregation Members*

searching for comfort and reassurance through the love and care of congregation members and clergy (an imam).

In the present study, we have not found informants who searched for comfort through the love and care of any imam or other religious leaders, but we did identify informants who received treatment from people who practice shamanism or alternative medicine. One 36-year-old woman explained this as follows:

There was one friend, who asked me not to do chemotherapy. He asked me to find alternative medicine. At that time, I just kept silent and smiled because I did get Islamic treatment alongside hospital treatment.

Another interviewee, a 33-year-old woman, informed us:

I went to seek spiritual treatment, I saw a religious person …. There was, according to him, someone who sent me the negative thing.

As in the case of religious coping through *Demonic Reappraisal*, here we are also dealing with the impact of culture more than that of religion. As it seems, the strong impact of shamanism and the shamanic view of health on people’s ways of thinking causes people to seek help through alternative treatment rather than through the spiritual guidance provided by imams.

## Discussion

As the present results indicate, some of the RCOPE methods seem to be highly relevant for the interviewees in Malaysia. As for our interviewees in Turkey (Ahmadi et al. 2016), for those in Malaysia, religion is a “larger part of [their] orientation system” (Ahmadi, 2006:28). Religion is, indeed, constantly available in people’s sociocultural context. Given this, it is understandable that, when facing a life-threatening illness like cancer, people turn to religion rather than to other resources. The present study shows, however, that for some interviewees, the role of culture in coping may be even stronger than that of fundamental religious axioms. Phenomena such as applying the coping method *Demonic Reappraisal*, believing in black magic and getting help from shamans/bomohs for alternative treatment reveal the strong role of cultural beliefs, even when they are in opposition to religious axioms. We discuss this point below.

Spiritual beliefs among Malay people are influenced by animism, Hinduism and Buddhism, all of which preceded the Islamization of Malaysia (Osman 1988). Concerning the Malay people’s indigenous knowledge about illness, some believe in witchcraft, black magic or *santau*, which originate from ancient mystical rituals. These ancient mystical rituals are believed to involve jinn and demons. They are used to destroy friendly relations among family members, to end spousal relationships, to bring about insanity and illness and, in the worst case, to cause the death of their victims (Mahyuddin 2014). Santau, which is a popular term among Malay people, is described as a poison that is sent using black magic or physically by the sender (Daud 2010). The motivation for sending *santau* is usually feelings of hatred and jealousy. It is believed that the illnesses caused by santau and black magic cannot be curing using conventional medical treatments (Sahad and Abdullah 2013); what is needed is religious treatment. *Ruqyah*, which refers to prayer therapy, is a common treatment among Malay people.

In the Malay world, shamans include pawang (specific kind of black or white magicians) or dukun and bomoh (spiritual counselors, traditional healers or medicine men). The bomoh’s original role was that of a healer. Before European colonization, bomoh is described as the most important person to integrate the complex beliefs and practices among traditional and rural communities (Osman 1989). These beliefs and practices were influenced by a combination of indigenous, Hindu and Islam that interacted and integrated to function in a whole. The bomoh’s craft remained largely unchanged even after Islam became dominant until the Islamic revival in the 1970s and 1980s. Bomohs were then seen as deviant from the Muslim faith because of their invocation of spirits and the potentially harmful black magic they were accused of practicing. This period saw a drastic decline in traditional herbalism, and many fraudulent practitioners filled the void (Sahad and Abdullah 2013). As a result, bomohs are today regarded with suspicion even though they are still commonly consulted by some Muslims.

In Malaysia, shamanism and cosmology are formed by a mixture of various elements of belief and religion, such as animism, Hinduism–Buddhism. Some shamans have tried to adapt their practice in the context of modern Islam, such as reciting verses of the Qur’an or invoking the name of Allah, but this practice is viewed as shallow by conservative shamans. The objection was also heard from Muslim leaders. The practice of bombo, as mentioned before, is regarded by Muslim scholars as *shirk* (idolatry, deification of figures other than Allah). *Shirk* is a major sin in Islam.

It is believed that the Malay originally believed in animism. This belief is still present in modern times among certain ethnic groups, such as the Senoi, Semang, Negrito, Kenak, Dayak. As Daud (2010: 183) maintains:

Their mystical views are replete with a variety of spirits connected with the forest, mountains, the sea, large trees, and hillocks and such things like. These spirits are an integral aspect of their lives and form a channel to realize their existence and their lives. Before the arrival of Islam approximately in the fourteenth century, peninsula Malays are said to have adhered to Hindu-Sivaism and subsequently to Hinayana Buddhism. The belief in the cosmos and the gods in these religions share with animism that they also believe that natural objects such as stones, hills, and the sun are endowed with power and spirit. This is an important aspect in Malay shamanism and mysticism. The Malay way of life and what they believed went through another change with the arrival of Islam, which brought the belief in the One and Only God (Allah), and with the Qur’an and hadith as guidelines in life. Slowly but surely, Hinduism and animism were set aside and were replaced by a belief system based on Islam. However, because animistic and Hindu elements were so firmly implanted in the Malay souls, Islam did not succeed in obliterating them completely. This is the reason these three elements form an integral part of Malay mysticism and shamanism. They colour their beliefs, values, and norms through fairytales that connect the real with the supernatural world and humans with the gods (Mohamed Ghouse Nasuruddin 2006: 10).

The interesting point here is, as mentioned above, the impact of the culture on health beliefs, which can even be stronger than fundamental religious axioms. According to Ahmadi (2015), culture is a basic element in construction of the belief system. The myths, symbols and rituals tied to religion can be seen as a way to try to understand the world. Cultural systems of heath explain what causes illness, how it can be cured or treated and who should be involved in the process. Applying *Demonic Reappraisal* as a coping method presumably requires belief in a Devil who can determine the course of individuals’ lives—a Devil who has the power to change a person’s “destiny.”

Indeed, in Islam, the devil is considered a creation of God and never becomes “evil as such”; he always remains a necessary instrument in God’s hand, because, in Islam, there is hardly any decisive dualism between good and evil or between God and Satan. The Qur’an does not portray Satan as an enemy of God, as God is greatest and supreme over all creation, Satan being just one of his creations. Satan’s only enemy is humanity.

Thus, a Muslim who strongly believes in an omnipotent God and does not regard Satan as possessing the power to change the course of events, in contrast to God’s will, can hardly redefine her/his stressor as an act of an evil power; everything is in the hands of God, not Satan. Therefore, shamanism was condemned often by religious leaders as shirk (idolatry) by referring to Qur’an, which directly condemns magicians and shamanists:^1^

… and the magician will never be successful, no matter what amount (of skill) he may attain (Taa Haa 20:69)

Despite this antipathy toward shamanism, the idea of black power seems to be quite strong in Malay culture (and generally among East Asian people), but it is more a superstition than a theological belief in the power of Evil. So here, we are witnessing the strength of old pre-Islamic cultural beliefs, which have survived even after Islam became predominant.

Summing up, the present study indicates that informants used several RCOPE methods, both passive and active. It also showed that shamanism—although it is in opposition to the religion of our informants (all Muslims)—plays a role in how they cope with cancer. The study highlights the important role of culture in the choice of coping methods. Following the present study, we are planning to conduct a quantitative survey study among cancer patients in Malaysia to examine the qualitative interview findings obtained here.

## Footnotes

1 Some Muslim groups, like some Sufi sects, have practiced shamanism, but Sufis have always been condemned and insulted by religious leaders and fundamentalists in many Islamic countries and some have even been executed; their ways of thinking can then hardly be considered predominant among Muslims. The influence of shamanism among some Muslim sects in the Middle East can be traced back to the thirteen century, when the Mongol empire conquered the Middle East. Shamanism was predominant among Mongols dating as far back as to the eighth century.
